# Mitochondrial Superoxide Dismutase in Cisplatin-Induced Kidney Injury

**DOI:** 10.3390/antiox10091329

**Published:** 2021-08-24

**Authors:** Kranti A. Mapuskar, Emily J. Steinbach, Amira Zaher, Dennis P. Riley, Robert A. Beardsley, Jeffery L. Keene, Jon T. Holmlund, Carryn M. Anderson, Diana Zepeda-Orozco, John M. Buatti, Douglas R. Spitz, Bryan G. Allen

**Affiliations:** 1 Free Radical and Radiation Biology Program, Department of Radiation Oncology, University of Iowa, Iowa City, IA 52242, USA; krantiashok-mapuskar@uiowa.edu (K.A.M.); emily-steinbach@uiowa.edu (E.J.S.); carryn-anderson@uiowa.edu (C.M.A.); john-buatti@uiowa.edu (J.M.B.); douglas-spitz@uiowa.edu (D.R.S.); 2 Biomedical Science Program, Holden Comprehensive Cancer Center, University of Iowa, Iowa City, IA 52242, USA; amira-zaher@uiowa.edu; 3 Galera Therapeutics, Inc., Malvern, PA 19355, USA; driley@galeratx.com (D.P.R.); rbeardsley@galeratx.com (R.A.B.); jkeene@galeratx.com (J.L.K.); jholmlund@galeratx.com (J.T.H.); 4 Center for Clinical and Translational Research, The Abigail Wexner Research Institute at Nationwide Children’s Hospital, Columbus, OH 43205, USA; diana.zepeda-orozco@nationwidechildrens.org; 5 College of Medicine, The Ohio State University, Columbus, OH 43210, USA; 6 Division of Nephrology, Department of Pediatrics, Nationwide Children’s Hospital, Columbus, OH 43205, USA

**Keywords:** superoxide dismutase, mitochondria, reactive oxygen species, mitochondrial metabolism, superoxide, cisplatin, acute kidney injury, chronic kidney disease

## Abstract

Cisplatin is a chemotherapy agent commonly used to treat a wide variety of cancers. Despite the potential for both severe acute and chronic side effects, it remains a preferred therapeutic option for many malignancies due to its potent anti-tumor activity. Common cisplatin-associated side-effects include acute kidney injury (AKI) and chronic kidney disease (CKD). These renal injuries may cause delays and potentially cessation of cisplatin therapy and have long-term effects on renal function reserve. Thus, developing mechanism-based interventional strategies that minimize cisplatin-associated kidney injury without reducing efficacy would be of great benefit. In addition to its action of cross-linking DNA, cisplatin has been shown to affect mitochondrial metabolism, resulting in mitochondrially derived reactive oxygen species (ROS). Increased ROS formation in renal proximal convoluted tubule cells is associated with cisplatin-induced AKI and CKD. We review the mechanisms by which cisplatin may induce AKI and CKD and discuss the potential of mitochondrial superoxide dismutase mimetics to prevent platinum-associated nephrotoxicity.

## 1. Introduction

Cisplatin is an inorganic, platinum-based agent used as a single agent or in combination with other agents for the treatment of a plethora of malignancies, including ovarian, cervical, head and neck, testicular, lymphoma, myeloma, small cell lung, and non-small cell lung cancers [1,2,3,4,5,6,7,8]. Cisplatin forms coordinate bonds with DNA purine bases; these crosslinks impair DNA repair mechanisms and thereby lead to cell cycle arrest and eventually cell death [9,10,11]. Cisplatin has been approved and utilized as an anti-cancer agent since the 1970s [12].

While the efficacy of cisplatin in several cancers is remarkably high, it is also well known for its acute and chronic side effects. Common adverse effects of cisplatin include nephrotoxicity, ototoxicity, myelosuppression, gastrointestinal symptoms, and neurotoxicity [10,13,14,15,16]. Cisplatin-induced acute kidney injury (AKI) is characterized by decreased renal function in conjunction with the accumulation of metabolic waste products such as urea and creatinine, whereas chronic kidney disease (CKD) is defined as kidney damage or glomerular filtration rate (GFR) <60 mL/min/1.73 m^2^ for 3 months or more, irrespective of cause according to Kidney Disease Quality Outcome Initiative (K/DOQI) [17,18,19]. Severe AKI is a known risk factor for CKD [20,21,22]. The use of cisplatin in cancer survivors is associated with an increased prevalence of CKD and CKD progression [23,24]. There is growing evidence to support a vital role of mitochondria in the process of cisplatin-induced renal toxicity through the generation of reactive oxygen species (ROS) both during AKI and CKD [25,26]. ROS is a collective term for oxygen-containing species generated during cellular metabolism. Increased levels of ROS can contribute to tubular cell apoptosis, as well as increased oxidative and nitrative stress, thereby causing kidney injury during AKI and CKD [27]. The mitochondrial electron transport chain (ETC) located on the inner mitochondrial membrane is a major source of ROS production in mammalian cells and tissues, with 0.1–1% of the electrons that flow through the ETCs expected to undergo one-electron reductions of oxygen forming superoxide (O_2_) and hydrogen peroxide (H_2_O_2_) [28,29]. Generation of ROS in tubular cell mitochondria may thus potentially contribute to cisplatin-induced kidney injury.

As highly dynamic organelles, mitochondria are involved in a variety of functions, including cellular signaling pathways, maintenance of redox metabolism, and ATP production [30,31,32,33]. Highly conserved dynamin-related GTPases act as the mediators of mitochondrial dynamics with frequent fission and fusion events. Dynamin-related protein 1 (Drp1) is involved in the process of mitochondrial fission, while OPA1 and mitofusins 1 and 2 (Mfn1 and 2) are required for mitochondrial fusion in mammalian cells [34]. Several cancer cell lines have demonstrated the potential role of mitochondrial dynamics in cisplatin resistance or sensitivity [35,36]. For example, studies in ovarian cancer cell lines treated with cisplatin resulted in mitochondrial morphology changes including punctate and fragmented mitochondria [30,37]. Increased mitochondrial fission mediated by Drp1 enhanced cisplatin sensitivity in ovarian cancer, leading to apoptosis [35]. OPA1-mediated mitochondrial fusion led to cisplatin resistance in neuroblastoma B50 rat cells [36]. Thus, the dynamic nature of mitochondria may potentially be utilized as a biomarker to predict cisplatin response.

## 2. Cisplatin as a Treatment Modality for Cancer

The use of cisplatin as an anticancer agent was first published in 1969, describing its action against malignant murine sarcoma and leukemia [38]. Higby and Wallace investigated cisplatin in metastatic testicular cancer, wherein they reported seven cases of complete recovery and 13 cases of significant tumor regression in a 15-patient clinical study [39]. Einhorn and Donohue combined cisplatin with bleomycin and vinblastine for advanced testicular cancer [40]. This three-drug regimen had an initial 70% complete re-sponse rate and five-year survival rate of 64% [40]. Wiltshaw and colleagues reported similar outcomes for advanced ovarian cancer using cisplatin as a single agent in 82 ovarian cancer patients previously treated with conventional chemotherapy [41]. Ovarian cancer response rate was dose-dependent, ranging between 33% for a 30 mg/m^2^ dose and 52% for a 100 mg/m^2^ dose [41]. On the basis of the success of these trials, cisplatin expanded to include additional malignancies such as cervical, lung, and head and neck cancers [34]. The outcomes were consistent with the testicular and ovarian cancer studies, wherein cisplatin was effective both as a single agent and in combination with other chemotherapeutic agents [42].

With cisplatin’s promising clinical trial success as an anti-cancer therapy, it became vital to understand this novel drug’s underlying mechanism of action. In 1970, Rosenberg and VanCamp proposed that cisplatin stimulated an immune response [43]. Later studies in mammalian cells and animals treated with cisplatin then revealed that the drug inhibits DNA synthesis and cell growth [44,45]. This discovery was made by tracing the incorporation of the radioactive DNA, RNA, and protein precursors 3H-thymidine, 3H-uridine, and 3H-L-leucine, respectively. Cisplatin hindered 3H-thymidine incorporation into DNA but not 3H-uridine or 3H-L-leucine incorporation both in vitro and in vivo [44,45]. It is now established that cisplatin binds to DNA purines at the N7 position and forms 1-, 2-, or 3-intrastrand crosslinks that terminate DNA replication and transcription and recruit high-mobility group box protein 1 (HMGB1), leading to the activation of pathways associated with DNA damage and apoptosis, such as p53 and MAPK [46].

Another noteworthy aspect of cisplatin’s history as an anti-cancer therapy is its radiation sensitizing activity. In 1978, Alvarez and colleagues reported that cisplatin sensitized TC.SV-40 cells against ionizing radiation in vitro [47]. As cisplatin showed efficacy as a chemotherapeutic agent in clinical trials, it was also tested in combination with radiotherapy. In 1981, 124 patients with advanced inoperable squamous cell carcinoma of the head and neck received cisplatin (100 mg/m^2^) every three weeks concurrently with definitive radiotherapy (planned total dose ≥ 64.5 Gy) [48]. Patients in this trial had significantly improved clinical response rates that differed on the basis of tumor site and differentiation state. Patients with hypopharyngeal cancer responded 25% of the time, while patients with nasopharyngeal tumors responded 83% of the time. The response rate for poorly differentiated tumors was 89% compared to 67% and 59% for well-differentiated and moderately differentiated tumors, respectively [46]. However, severe toxicities associated with this treatment regimen included leukopenia (11%), nausea and vomiting (8%), stomatitis (31%), and nephrotoxicity (6%) [48].

Subsequent randomized clinical trials have shown concurrent cisplatin improves locoregional control, progression-free survival, and overall survival in non-small cell lung cancer [49], cervical cancer [50], and head and neck cancer [51,52,53] over radiation alone, induction chemotherapy, or radiation in combination with other agents. Rates of severe toxicities from concurrent cisplatin in these trials include leukopenia (11–42%), nausea and vomiting (8–28%), stomatitis (31–43%), anemia (17%), dermatitis (7%), neurologic toxicity (5%), and nephrotoxicity (4–8%) [48,49,50,51,52,53]. Any grade acute kidney injury incidence is as high as 34% with high dose cisplatin (100 mg/m^2^ q3 weeks) [54]. The risk of cisplatin-induced nephrotoxicity increases with cisplatin dose and duration of treatment [55]. For example, 34% of head and neck cancer patients treated with fractionated ionizing radiation (total dose of 60 to 70 Gy in 2 Gy fractions) and cisplatin therapy (100 mg/m^2^ delivered every 21 days for 3 cycles) develop cisplatin-induced AKI [54]. A decline in renal function may necessitate cisplatin administration delays and dose reductions as patients cannot receive a planned dose of cisplatin [54]. Risk factors for developing nephrotoxicity following cisplatin exposure are related to the renal clearance of cisplatin. Patients prone to developing AKI following cisplatin treatment include those that have high peak plasma cisplatin concentrations (>400 ng/mL) [52], pre-existing kidney damage (creatinine > 1.5 mg/dL) [53], age ≥ 61 years, and a history of hypertension [56,57]. Survivors of childhood cancers treated with cisplatin (cumulative doses > 450 mg) develop long-term (decades) nephrotoxicity with reduced estimated glomerular filtration rates compared to childhood cancer survivors not treated with cisplatin (eGFR of 83 mL/min/1.73 m^2^ vs. 101 mL/min/1.73 m^2^). Adult cancer survivors treated with cisplatin are also prone to worsening long term renal function and chronic kidney disease. A retrospective review of 777 adult cancer survivors treated with cisplatin had an average eGFR reduction of 0.73 mL/min per 1.73 m^2^ per year.

RTOG-1016 randomized 849 subjects with locally advanced oropharyngeal carcinoma to receive radiotherapy (70 Gy/35 fx) combined with either cisplatin (100 mg/m^2^ on days 1 and 22 of radiation) or cetuximab (loading dose of 400 mg/m^2^ for 5–7 days followed by weekly cetuximab at 250 mg/m^2^ for seven doses). Patients treated with cisplatin had an improved 5-year progression-free survival (78% vs. 67%) and reduced 5-year local regional failure (9.9% vs. 17%). There were no significant differences in xerostomia, fibrosis, muscle atrophy, and weight loss. On the basis of these data, the research found that radiation combined with cisplatin is superior to radiation combined with cetuximab for the definitive treatment of locally advanced oropharyngeal carcinoma [52].

Despite its treatment efficacy, cisplatin treatment is known to cause significant toxicities. A phase III intergroup trial in head and neck cancer patients comparing subjects that received radiation alone (70 Gy/35 fx), radiation and cisplatin (100 mg/m^2^ on days 1, 22, and 43), or split course radiation was given with three cycles of 5-fluorouracil and cisplatin chemotherapy, identifying improved 3-year overall survival in patients treated with concurrent cisplatin and radiation [48]. Relative to subjects receiving radiation alone, however, subjects treated with concurrent cisplatin and radiation had an increased risk for ≥ grade 3 nausea and vomiting (16% vs. 6%), leukopenia (42% vs. 1%), anemia (17% vs. 0%), and nephrotoxicity (8% vs. 1%).

A retrospective review of 821 adult cancer survivors treated with cisplatin who survived for at least 5 years demonstrated the following changes in renal function: patients who were CKD stage 1 pre-cisplatin treatment progressed to CKD stage 2 (48%) or CKD stage 3 (14%), while only 36% remained at CKD stage 1 [23].

A common clinical approach to prevent and reduce the severity of cisplatin-associated nephrotoxicity is pre-hydration with intravenous isotonic saline to increase diuresis [58]. Additional common clinical approaches include avoiding concomitant nephrotoxic drugs, reducing cisplatin dose [59], and substituting an alternative chemotherapy agent for cisplatin [60]. Examples of additional approaches that are less commonly utilized clinically include amifostine and theophyilline. Amifostine is approved by the FDA to reduce renal injury associated with multiple cisplatin administrations [61,62]. Amifostine is a thiol derivative that scavenges free radicals generated during radiation and chemotherapy [63]. Pre-clinical studies demonstrate that amifostine reduces mitochondrial membrane potential and reactive oxygen species formation in murine hepatocytes but not in hepatoma cells [64]. However, because amifostine has a short half-life and significant side effects (nausea, vomiting, and hypotension), it is rarely used clinically [61]. Theophylline is a competitive inhibitor of the adenosine receptor [65]. Adenosine reduces GFR by constricting afferent arterioles, and preclinical studies demonstrated that adenosine receptor antagonists reduced acute renal injury [66,67]. A randomized, single-blinded, placebo-controlled trial in 41 patients receiving cisplatin (50 mg/m^2^) as part of their chemotherapy regimen demonstrated that theophylline preserved GFR compared to placebo-controlled subjects [65].

Whether as a single chemotherapeutic agent, in combination with other chemotherapies, or in combination with ionizing radiation, cisplatin is still considered one of the most essential and reliable treatment agents for numerous malignancies. However, cisplatin-associated toxicities, especially nephrotoxicity, can dramatically hinder individual patient clinical outcomes; therefore, research dedicated to understanding and overcoming cisplatin toxicity is critical.

## 3. Characterization of Kidney Injury

The Kidney Disease: Improving Global Outcomes (KDIGO) guidelines define AKI as an abrupt decrease in kidney function that occurs over a period of 7 days or less, and CKD as abnormalities in kidney structure or function that persist for >90 days [19,68]. Acute kidney disease (AKD) is described by KDIGO as acute or subacute damage or loss of kidney function for a duration of between 7 and 90 days after exposure to an AKI-initiating event [19,68]. Several definitions of AKI have been validated, including the risk, injury, failure, loss of kidney function, and end-stage kidney disease (RIFLE) classification based on serum creatinine (sCr) or urinary outputs (UO) (Table 1) [69], with the acute kidney in-jury network (AKIN) classification being based on a ≥50% increase in absolute sCr (1.5 × baseline value) or a decrease in UO to <0.5 mL/kg/h for more than six hours [69]. The AKIN classification uses the staging system described in Table 1. After diagnosis of AKI by either classification, the KDIGO guidelines suggest monitoring sCr and UO for three months for resolution, new-onset, or worsening kidney dysfunction leading to chronic kidney disease (CKD) [54]. Criteria to meet the definition of CKD is determined by duration; glomerular filtration rate (GFR); and abnormal urinalysis, pathology, or structure of the kidneys [70]. CKD staging is based on GFR (mL/min/1.73 m^2^) and the presence of albuminuria (Table 2). Hypertension, diabetes, and hypercholesterolemia are risk factors for the development of CKD. Current CKD staging is based on GFR (mL/min/1.73 m^2^) and presence of albuminuria (Table 2). Long-term kidney dysfunction is notable in 60–80% of patients who receive cisplatin chemotherapy [71].

## 4. Pathophysiology of AKI and CKD

While the term “AKI” is clinical, the use of acute tubular injury (ATI) is used to classify kidney injury histopathologically. In practice, ATI is semi-quantified as either mild, moderate, or severe injury and as well as focal vs. diffuse injury [72]. Characterization of kidney biopsy samples for ATI is made by the presence of tubular luminal dilation, loss of the brush border in tubules, loss of nuclei, and the presence of cytoplasmic basophilia [72]. Additionally, distinct pathological markers can be found in AKI associated with pigment administration, crystallopathy, nephrotoxic drug administration, and infection [72]. An increase in pathophysiology studies has revealed that oxidative stress, endothelial injury, mitochondrial injury, and immunological responses are key mechanisms to the AKI development of AKI. Furthermore, AKI is now considered a prominent risk factor for the CKD development of CKD, particularly in older patients and patients who have had multiple AKI episodes [72].

The definition of CKD includes not only decreases in GFR, but also structural and functional abnormalities of the kidney. Functional abnormalities such as albuminuria, proteinuria, and hematuria are classic examples. Glomerular filtration is highly dependent on high intra- and trans-glomerular pressure, which is reflected in hemodynamic injury to the kidney [73]. Additionally, CKD is promoted when the glomerular membrane’s electrostatic barrier is disrupted, allowing proteins to move into Bowman’s capsule [73]. Tubulointerstitial impairment also closely associates with long-term kidney dysfunction and encompasses many pathological features such as interstitial inflammation, kidney fibrogenesis, fibroblast activation, and promotion of the epithelial–mesenchymal transition (EMT) [73].

## 5. Mechanism of Cisplatin-Induced Kidney Injury

### 5.1. Accumulation

**Figure 1:**
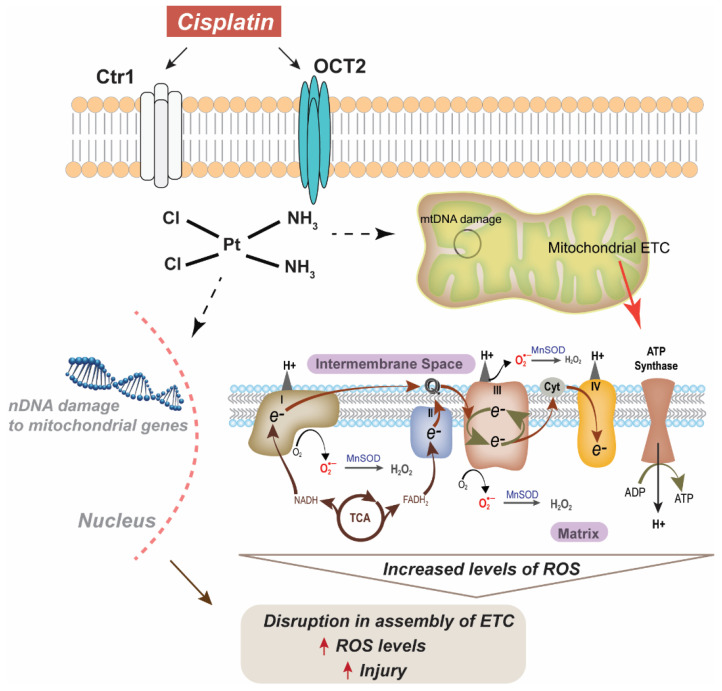
Scheme for cisplatin-induced injury via damage to both nuclear (nDNA) and mitochondrial DNA (mtDNA). Ctr1: copper transporter 1, OCT2: organic cation transporter 2, MnSOD: manganese superoxide dismutase, ROS: reactive oxygen species, ETC: electron transport chain.

Cisplatin uptake in the kidney is relatively unstudied and may vary between cell types. The organic cation transports (OCTs) have been implicated in the transport of cisplatin from the basolateral to the apical side in tubular cells [73,74] (Figure 1). While three isoforms of the OCT are found in the kidney, OCT2 has been found to be the largest transporter of cisplatin [73]. Upregulation of OCT2 has been shown to correlate with magnesium deficiency, which subsequently promotes the intratubular intake of cisplatin. This magnesium deficiency concurrently downregulates the multi antimicrobial extrusion protein 1 (MATE1), which is expressed at the brush-border membrane in proximal tubular cells, limiting cisplatin outtake. The combined effect of OCT2 upregulation and decreased MATE1 expression enhances cisplatin-induced AKI [75]. After cisplatin enters the tubule cells, it may undergo a variety of metabolic activations. Common pathological findings in cisplatin-treated kidney tissues are tubular cell death, apoptosis, and necrosis. Apoptosis and necrosis share similar signaling pathways, including those involved in the mitochondrial damage pathway [73]. Previous studies have shown that kidney mitochondria are the primary targets for cisplatin toxicity and that mitochondrial DNA damage drives cisplatin nephrotoxicity [25,76,77]. Mitochondria stressed by cisplatin activate caspase-mediated apoptosis by the release of caspase-9 activators. Mitochondrial DNA is also a prime target for platinum crosslinking due to the lack of efficient mitochondrial DNA repair mechanisms. This DNA is critical for encoding several inner membrane proteins including cytochrome-c oxidase subunits and ATPase [76]. Cytochrome-c oxidase (COX, complex IV) generates the proton motive force, which drives ATP production. Recent studies have shown COX enzymatic activity is weakened in proximal tubule epithelium after cisplatin treatment [78]. Furthermore, it has also been reported that this decrease in COX activity is partially due to a decrease in mitochondrial mass [79]. An early feature associated with cisplatin nephrotoxicity is oxidative stress presenting as increased 4-hydroxy-2-nonenal and increased nitro tyrosine content in mitochondrial extracts [79]. Additionally, abnormal lipid peroxidation and disruption to the synthesis of adenosine triphosphate (ATP) result in the aberrant production of free radicals and ROS.

### 5.2. Metabolism

Once in the kidney, cisplatin is metabolized to its active form, which is a renal toxin via a platinum-glutathione conjugate to a reactive sulfur-containing compound. This platinum-cysteine S-conjugate is bio-transformed into a reactive thiol by a pyridoxal 5’-phosphate-dependent cysteine S-conjugate β-lyase [80]. The platinum–glutathione conjugate is cleaved to a platinum–cysteinyl–glycine conjugate by gamma-glutamyl transpeptidase (GGT) on the cell surface and is subsequently cleaved to a platinum–cysteine conjugate by a dipeptidase [81,82]. The platinum–cysteine conjugate is then taken up into the cell, where it is converted to a highly reactive thiol by cysteine S-conjugate-lyase. The reactive thiol binds to cellular proteins that induced apoptosis, thereby contributing to AKI [80,83].

While the transition of AKI to CKD has yet to be illustrated, tubular cell death, oxidative distress, and vascular injury are some other mechanisms that contribute to the AKI to CKD transition in cisplatin-treated patients [73]. Further investigation into cisplatin’s nephrotoxic pathways is needed.

## 6. Mitochondria in Cisplatin-Induced Injury

The widespread use of cisplatin as a chemotherapeutic drug is based on the mechanism that cisplatin forms adducts to nuclear DNA (nDNA), thereby inducing cell death [84]. Interestingly, in addition to nDNA, cisplatin also affects mitochondrial DNA (mtDNA). Yang et al. in 2006 showed that cisplatin adducts to mtDNA were 300–500-fold more abundant than adducts to nDNA and that these differences were not based on the rate of adduct repair [84]. Studies have also shown that mtDNA is also susceptible to cisplatin-induced inhibition of replication and that cisplatin inhibits the transcription of mitochondrial genes [85]. The significance of mitochondrial metabolism in cisplatin induced injury is evident from the fact that cells depleted of their mitochondrial DNA (rho0 cells) show decreased cisplatin-induced cytotoxicity as compared to the parental cell line [86]. Furthermore, studies also suggest that cells respond to cisplatin by increasing mitochondrial content, which correlates to cisplatin-induced apoptosis [87].

### 6.1. Cisplatin-Induced Changes in Mitochondrial Morphology and Bioenergetics

Structural and functional changes to the mitochondria have also been noted following treatment with cisplatin [25]. Treatment of RTECs with cisplatin decreases mitochondrial mass along with changes in mitochondrial morphology (disruption of cristae and excessive mitochondrial swelling) and reductions in mitochondrial activity and ATP production [78,79]. Cisplatin-induced peripheral neuropathy studies in rodents have demonstrated abnormal mitochondrial morphology including disorganized cristae and disrupted double membranes [88]. Furthermore, the study also indicated an accumulation of mitochondrial p53 preceding an acute hypo polarization of mitochondria (decreased mitochondrial membrane potential) 4 h following treatment with a single dose of 2.3 mg/kg cisplatin [88]. Studies have also identified an association between a cell’s mitochondrial density and its sensitivity to cisplatin-induced cell death. Since renal tubular epithelial cells (RTECs) have the highest mitochondrial density compared to the rest of the nephron, this abundance may be the key to cisplatin’s preferential damage to RTEC mitochondria [89].

### 6.2. Redox Homeostasis

The mitochondrial dysfunction induced by treatment with cisplatin can be characterized by increased steady-state levels of reactive oxygen species (ROS), hypo-polarization (reduced mitochondrial membrane potential), and ATP depletion, resulting in cellular apoptosis [90]. ROS are generated as a by-product of normal cellular metabolism in the mitochondria and the cytoplasm in small amounts; however, excessive ROS production can prove detrimental and thus lead to injury. It is estimated that in mammalian mitochondria, 0.1–1% of O_2_ consumption could potentially result in ROS formation (O_2_^•−^ and H_2_O_2_) [91,92,93]. In addition to mitochondrially generated ROS, several other metabolic processes could also contribute to ROS production, including NADPH oxidase enzymes, cytochrome P450 enzymes, xanthine oxidase, and several peroxisomal enzymes [91,92,93]. Increased ROS production is known to alter the mitochondria membrane potential and thus the electron transport chain, further exacerbating ROS levels and ultimately leading to apoptosis [94,95,96]. Murine studies have also demonstrated disruptions in mitochondrial metabolism that lead to increased levels of mitochondrial superoxide following treatment with cisplatin, which may play an important role in AKI and CKD [25].

### 6.3. Antioxidant Defense

In addition to increased ROS levels, treatment with cisplatin also impairs the activity of antioxidants that function to regulate ROS levels, such superoxide dismutase (SOD), catalase (CAT), and glutathione peroxidase (GPx) [97]. This results in impairment of the antioxidant system following cisplatin treatment, which can lead to oxidative stress. Once in the renal epithelial cells, cisplatin is converted into a nephrotoxin (as discussed above) via the metabolic activation of GGT. The highly reactive thiol molecule produced after cisplatin uptake is metabolized by cysteine S-conjugate-lyase, increasing ROS levels [10]. Cisplatin treatment results in persistent upregulation of kidney injury markers including neutrophil gelatinase-associated lipocalin (NGAL) and kidney injury marker-1 (KIM-1), increased steady-state levels of O_2_, increased tubule damage, and upregulation of mitochondrial electron transport chain (ETC) complex I activity up to one month following cisplatin treatment, suggesting a pivotal role for mitochondria in the injury process [25]. Furthermore, treatment with cisplatin has been shown to decrease the protein expression of mitochondrial ETC complexes (C I, III, IV), MnSOD, and glutathione levels, thereby indicating a decline in mitochondrial ETC function in the kidney [98]. Therefore, targeting the cisplatin-induced oxidative stress via manipulation of the cellular antioxidant system could be beneficial for protecting against cisplatin nephrotoxicity.

Another pathway that has been extensively explored in cisplatin resistance is the NRF2 pathway. As mentioned before, the cytotoxic effect of cisplatin is primarily due to its ability to bind to DNA, thus causing increased levels of DNA damage. An increase in DNA repair capacity is thus the most apparent way to increase resistance to cisplatin and as such has been the focus of several studies. One such mechanism involves increase in intracellular levels of glutathione (GSH), which is a prevalent cellular antioxidant. GSH can bind and thus inactivate cisplatin via its reactive thiol group, preventing DNA damage [99]. The enzymes responsible for GSH synthesis and utilization, including the glutamate-cysteine catalytic subunit (GCLC) and glutamate-cysteine ligase modifier subunit (GCLM), heme oxygenase 1 (HO-1), glutathione peroxidase, glutathione reductase, and glutathione-S-transferase (GST), are regulated by nuclear factor, erythroid-derived 2-like 2 factor (NRF2), which is the master regulator of antioxidant response [100].

Under non-stressed conditions, NRF2 is bound to KEAP1 and promotes its ubiquitination and proteasomal degradation. Under conditions of oxidative stress, Keap1-dependent Nrf2 degradation mechanism is inactivated, and NRF2 translocates to the nucleus and promotes the transcription of several antioxidant response element (ARE)-dependent cytoprotective genes. Overexpression of NRF2 has been recently correlated to cisplatin resistance in different types of cancer [99,101,102]. To overcome this resistance to cisplatin, studies have used genetic tools such as small interfering RNA (siRNA) against NRF2 to promote the production of ROS following cisplatin treatment, leading to cisplatin-induced sensitivity in cancer cells [103]. Additionally, other strategies to induced NRF2 expression have been used in cancer such as somatic mutations in NRF2 (gain of function mutation) and epigenetic alterations in KEAP1 that leads to aberrant activation and nuclear translocation of NRF2 [104,105,106]. Other studies have used pharmacological manipulations to inhibit NRF2 such as treatment with natural flavonoids, polyphenol conjugates, phytochemicals, and alkaloids [107,108,109,110]. Thus, modulation of the NRF2 pathway has been considered as a promising strategy to overcome cisplatin resistance in several cancers.

## 7. Mitochondrial Superoxide Dismutase and Cisplatin-Induced Kidney Injury

Superoxide dismutases (SOD) are ROS-detoxifying enzymes with various subcellular localizations, including copper-zinc SOD (CuZnSOD, SOD1), a homodimer primarily localized to the cytoplasm and the mitochondrial intermembrane space [111]; extracellular SOD (ECSOD, SOD3), localized to the extracellular regions of the cell [112]; and manganese SOD (MnSOD), localized to the inner mitochondrial membrane. MnSOD is the primary mitochondrial enzyme that catalyzes the dismutation of superoxide (formed due to one-electron reduction of oxygen) to hydrogen peroxide and molecular oxygen [113]. MnSOD is a nuclear encoded protein that is transported into the mitochondria via an amino-terminal targeting sequence and subsequently cleaved, forming its native homo-tetrameric structure of 96 kDa [114]. Homozygous deletion of MnSOD is embryonically lethal, resulting in metabolic acidosis, cardiomyopathy, and neurodegeneration [115,116,117,118,119]. MnSOD heterozygous mice are susceptible to oxidative damage, suggesting a central role of MnSOD in mitochondrial metabolism and function [115,116,117,118,119]. The electron transport chain (ETC) is located on the inner mitochondrial membrane and is likely the primary source of ROS generation. Mitochondrial ETC dysfunction resulting in delayed electron transport may increase ROS generation and is proposed to cause a variety of conditions, including cisplatin-induced kidney injury. Kidney, being one of the most energy-demanding organs, has a high resting metabolic rate and the second highest mitochondrial content and oxygen consumption rates following the heart (PMID: 28804120). The abundance of mitochondria in the kidney and mitochondria being a major source of ROS makes kidney susceptible to cisplatin-induced injury. Thus, the prime focus of recent studies has been therapeutic manipulations targeting mitochondrial antioxidant levels to alleviate the effects on kidney injury. To this end, several mitochondria-based studies support the hypothesis that the overexpression of mitochondrial antioxidant enzymes (MnSOD) or treatment with MnSOD mimetics should interrupt the injury process, thereby alleviating some of the effects of cisplatin treatment.

Studies have shown that overexpression of MnSOD in HEK293 (Human Embryonic Kidney 293) cells increased cloning efficiency and decreased DNA fragmentation and annexin V levels following treatment with cisplatin [120]. Furthermore, overexpression of MnSOD protected HEK293 cells from cisplatin-induced cytotoxicity as indicated by decreased cell rounding, detachment, and cell size [120]. As a result of disruptions in mitochondrial metabolism, persistent increases in superoxide levels mediate cisplatin-induced chronic kidney disease [25]. Inactivation of MnSOD has also been shown to release pro-apoptotic factors including cytochrome C, loss of mitochondrial membrane integrity, and a decrease in the production of ATP following renal injury [96]. Treatment with cisplatin has also been shown to decrease MnSOD expression, increase mitochondrially generated ROS, and in-duce cell death in HK2 cells, which was mitigated following treatment with MnTBAP, a MnSOD mimetic [121]. A recent study using a murine model of cisplatin induced AKI demonstrated that Sirtuin 3 knockout mice experience severe AKI compared to their WT counterparts with increased kidney dysfunction and decreased survival [122]. Sirtuin 3 (Sirt3) is a mitochondrial deacetylase that removes acetyl groups from lysine residues on specific protein targets including MnSOD [123], catalase [124], and glutathione peroxidase [125]. Post-translation modification of MnSOD via acetylation changes the function of MnSOD from a superoxide scavenging homo-tetramer to a peroxidase-directed monomer [126]. Furthermore, acetylation of MnSOD is detrimental to mitochondrial metabolism by increasing superoxide levels and altering mitochondria structure and function in cisplatin-resistant breast cancer cell lines, suggesting a pivotal role of MnSOD as a regulator in cisplatin-induced injury [127].

## 8. Dismutase Mimetics and Cisplatin-Induced Kidney Injury

Compared to other kidney cells, RTECs are heavily damaged following cisplatin administration [25]. RTECs have increased mitochondria content compared to other kidney cells [128]. Because mitochondria ETC are major sources of ROS formation, disruption of RTEC ETC makes them especially susceptible to cisplatin-induced injury. Alterations in the ETC and dysfunctional mitochondrial metabolism may lead to increased oxidative stress, apoptosis, and fibrosis, which can all compromise overall renal function.

Limited data are available on studies that focus on using compounds specifically targeting mitochondria as a potential therapeutic strategy to prevent AKI and CKD. Tempol (4-hydroxy tempo) is a non-specific ROS scavenger that is membrane-permeable and can scavenge both superoxide and hydrogen peroxide [129]. Pre-treatment of albino mice with 100 mg/kg tempol daily for four days followed by a single dose of cisplatin (25 mg/kg) significantly decreased the incidence of cisplatin-induced kidney injury as assessed by changes in serum creatinine, serum urea, glucosuria, and proteinuria [129]. Furthermore, pre-treatment with Tempol prevented the decreased ETC complex I and III activities and ATP levels seen with cisplatin administration. These data suggest that pharmacologically scavenging ROS may lessen cisplatin-induced mitochondrial dysfunction and thereby minimize cisplatin-induced kidney injury.

Avasopasem (previously known as GC4419) is a small-molecule, selective dismutase mimetic developed by Galera Therapeutics Inc. that is currently in phase 3 clinical trials for radiation-induced oral mucositis in head and neck cancer patients receiving radiation (70 Gy/35 fx) (NCT03689712). GC4419 at 90 mg before each radiation fraction significantly reduced the duration, incidence, and severity of severe oral mucositis (SOM) [130,131,132]. Preclinically, GC4419 has also been shown to abrogate both the age-associated disruptions in mitochondrial ETC function and enhanced sensitivity to radiation and cisplatin-induced injury while not reducing radiation and chemotherapy-induced tumor cell killing [25,26] and even enhancing the anti-cancer effects of hypo-fractionated radiation [105]. Previously published preclinical data from our group has shown that treatment with GC4419 ameliorates cisplatin-induced kidney injury as assessed by changes in BUN and serum creatinine, restores cisplatin associated reductions in ETC complex I activity, and minimizes mitochondrial morphology changes due to increases in O_2_ [25].

## 9. Conclusions

These studies indicate that changes in mitochondrial oxidative metabolism along with altered expression of antioxidants, including SODs, and resulting increased mitochondrial ROS are all crucial in cisplatin-induced AKI and CKD. Thus, manipulations of mitochondrial ROS and ETC via increasing MnSOD activity may potentially minimize cisplatin-induced kidney injury. Protection of kidneys from cisplatin-induced injury might be achievable by combining cisplatin-based drugs with antioxidant-based therapeutic interventions that increase antioxidant levels and thus mitigate ROS damage while maintaining anti-cancer efficacy. These therapeutic approaches may enhance the tolerance to cisplatin and hence enable greater dose intensity associated with better outcomes.

## Figures and Tables

**Table 1:** AKIN vs. RIFLE classification for kidney injury based on serum creatinine (sCr) and/or urinary outputs (UO).

AKIN	UO (Common to Both)	RIFLE
Stage 1 Increase of ≥ 0.3 mg/dl or increase in more than or equal to 150–200% from baseline.	Less than 0.5 mg/kg/L per hour for more than 6 h	Risk Increase in sCr × 1.5 or GFR decrease >25%
Stage 2 Increase to more than 200–300% from baseline.	Less than 0.5 mg/kg/L per hour for more than 12 h	Injury sCr × 2 or GFR decrease >50%
Stage 3 Increased to more than 300% from baseline with an acute increase of at least 0.5 mg/dL or on RRT.	Less than 0.3 mg/kg/L for 24 h or anuria for 12 h	Failure sCr × 3 or >4 mg/dL with an acute rise >0.5 mg/dL or GFR decrease >75%
		Loss Persistent acute kidney failure = complete loss of kidney function >4 weeks
		End-Stage Kidney Disease ESKD >3 months

AKIN, Acute Kidney Injury Network; ESKD, end-stage kidney disease; GFR, glomerular filtration rate; sCr, serum creatinine; RIFLE, risk, injury, failure, loss, and end stage; RRT, renal replacement therapy.

**Table 2:** Staging system for chronic kidney disease as per Kidney Disease Improving Global Outcomes (KDIGO) guidelines.

GFR Stages	Kidney Function	GFR (mL/min/1.73 m^2^)
Stage G1	Normal	≥90
Stage G2	Mildly Decreased	60–90
Stage G3a	Mildly to Moderately Decreased	45–59
Stage G3b	Moderately to Severely Decreased	30–44
Stage G4	Severely Decreased	15–29
Stage G5	Kidney Failure	<15
